# Prion Amplification and Hierarchical Bayesian Modeling Refine Detection of Prion Infection

**DOI:** 10.1038/srep08358

**Published:** 2015-02-10

**Authors:** A. Christy Wyckoff, Nathan Galloway, Crystal Meyerett-Reid, Jenny Powers, Terry Spraker, Ryan J. Monello, Bruce Pulford, Margaret Wild, Michael Antolin, Kurt VerCauteren, Mark Zabel

**Affiliations:** 1 Colorado State University Prion Research Center, Department of Microbiology, Immunology and Pathology, College of Veterinary Medicine and Biomedical Sciences, Fort Collins, CO 80523, USA; 2 National Wildlife Research Center, Wildlife Services, United States Department of Agriculture, La Porte Avenue, Fort Collins, CO 80521; 3 Department of Biology, College of Natural Sciences, Colorado State University, Fort Collins, Co 80523; 4 National Park Service, Biological Resource Management Division, 1201 Oakridge Drive, Fort Collins, CO 80525, USA

## Abstract

Prions are unique infectious agents that replicate without a genome and cause neurodegenerative diseases that include chronic wasting disease (CWD) of cervids. Immunohistochemistry (IHC) is currently considered the gold standard for diagnosis of a prion infection but may be insensitive to early or sub-clinical CWD that are important to understanding CWD transmission and ecology. We assessed the potential of serial protein misfolding cyclic amplification (sPMCA) to improve detection of CWD prior to the onset of clinical signs. We analyzed tissue samples from free-ranging Rocky Mountain elk (*Cervus elaphus nelsoni*) and used hierarchical Bayesian analysis to estimate the specificity and sensitivity of IHC and sPMCA conditional on simultaneously estimated disease states. Sensitivity estimates were higher for sPMCA (99.51%, credible interval (CI) 97.15–100%) than IHC of obex (brain stem, 76.56%, CI 57.00–91.46%) or retropharyngeal lymph node (90.06%, CI 74.13–98.70%) tissues, or both (98.99%, CI 90.01–100%). Our hierarchical Bayesian model predicts the prevalence of prion infection in this elk population to be 18.90% (CI 15.50–32.72%), compared to previous estimates of 12.90%. Our data reveal a previously unidentified sub-clinical prion-positive portion of the elk population that could represent silent carriers capable of significantly impacting CWD ecology.

CWD is a neurodegenerative disease first seen in captive Colorado cervid populations in 1967, later identified as a transmissible spongiform encephalopathy (TSE) in 1978^1^ and first found in free-ranging populations in 1981^1^,^2^. Prions, the infectious agent of TSEs, arise from the misfolding of the normal host-encoded cellular prion protein (PrP^C^) into an insoluble, aggregated and infectious form that resists protease degradation. Prions causing CWD arise from PrP^CWD^, the misfolded, infectious form of cervid PrP^C^. CWD, the only known TSE to occur in free-ranging wildlife, affects several cervid species including elk (*Cervus elaphus nelsoni*), mule deer (*Odocoileus hemionus*), white-tailed deer (*O. virginianus*) and less commonly moose (*Alces alces spp.*). Prevalence of CWD in free-ranging deer populations in Colorado and Wyoming ranges from 0–30% and prevalence in free-ranging elk herds ranges from 0–13% in Colorado^3^,^4^,^5^,^6^. CWD and sheep scrapie are capable of both horizontal transmission from infected individuals^7^,^8^,^9^ as well as efficient indirect transmission from contaminated environments^10^,^11^,^12^,^13^.

Despite cross-species transmissibility of PrP^CWD^ among cervids, the pathogenesis of the disease may be different between deer and elk. For example, studies with experimentally infected deer suggest that PrP^CWD^ infect and replicate in peripheral lymphatic tissues prior to neuroinvasion^14^,^15^. Studies with captive or experimentally infected elk are less conclusive and suggest prions may first be detectable in the obex of the brain stem or the retropharyngeal lymph node without a clear pattern^16^,^17^,^18^. Studies of free-ranging elk, however, suggest that PrP^CWD^ can likely be detected first in lymphatic tissue^19^. It is unclear if route of inoculation or prion strain can also affect these apparent differences.

Infected animals shed prions into the environment through saliva, feces, urine and even antler velvet^15^,^20^,^21^,^22^,^23^,^24^. Studies have successfully transmitted PrP^CWD^ through a single dose of urine or feces from animals displaying signs of CWD, indicating that at the time of clinical disease sufficient prions are shed to result in an infectious dose^24^,^25^. However, at what stage(s) of disease animals shed prions into the environment remains unclear. If shedding occurs early in disease, a sub-clinical animal may not only shed prions into the environment, increasing the infectious reservoir, but may also transmit CWD horizontally to their associates. Unfortunately little is known about the prevalence of early or sub-clinical infection, and what role they may play in CWD transmission ecology. This ignorance raises a critical question that must be answered: how long do free-ranging animals live once infected? Answering it requires detection of prion infections as early as possible in the course of infection.

Cervid prion protein gene (PRNP) polymorphisms can influence CWD kinetics. Elk PRNP methionine or leucine polymorphism at codon 132 can dramatically affect incubation time and possibly susceptibility of inoculated elk^20^,^21^,^22^,^23^,^24^,^26^. Experimental and observational evidence suggests that 132LL homozygous elk are rare (≤2.5%) in free-ranging populations^20^,^25^,^27^,^28^, and when inoculated have a substantially delayed disease course (>48 months) in captivity compared to MM homozygote and ML heterozygote elk (12–24 months)^26^,^29^. Despite differences in disease kinetics, Perucchini et al.^27^ found that the prevalence of CWD within elk genotypes was not disproportionate, indicating the three genotypes maintain proportional CWD prevalence despite the disease course differences. Whether cervid PRNP genotype affects transmission and shedding of PrP^CWD^ remains uncertain. Surveys of free-ranging deer suggest increased rates of CWD between 2–11 years of age^30^ but work on elk suggests a much wider age range can be infected^19^ and no study to date has quantified the interaction between age and prevalence of CWD in elk.

Currently, prion protein immunohistochemistry (IHC) of brain tissue and lymph nodes is the gold standard for CWD detection^31^. However, this method requires 2–3 weeks for sample preparation, relatively large quantities of well-preserved tissue and specialized training for accurate microscopy work. Commercially available enzyme-linked immunosorbent assay-based tests detect prion infection more rapidly, and with similar sensitivity to IHC^32^. Serial protein misfolding cyclic amplification (sPMCA) has emerged in the field of prion research as a reliable and sensitive alternative detection assay for a variety of tissue and sample types^1^,^22^,^27^,^33^,^34^,^35^,^36^,^37^. Haley et al.^38^ found comparable sensitivity between an abbreviated sPMCA vs. IHC in longitudinal tonsil biopsies from experimentally inoculated, captive white-tailed deer.

We have optimized both sensitivity and specificity to maximize PrP^CWD^ amplification and detection, leading to a more sensitive sPMCA protocol that processes samples in less than two weeks^23^. We used sPMCA to test obex tissue samples from a free-ranging elk herd in the Rocky Mountain National Park (RMNP), Estes Park, CO; and Bayesian hierarchical modeling to compare our results to IHC of retropharyngeal lymph node and obex tissue and estimate prevalence of PrP^CWD^ in this herd.

## Results

Of the 85 elk tested, 20 were IHC-positive in one or more tissues (Table 1). Of the 20 IHC-positive animals, sPMCA identified 19 correlating obex samples as positive. The one sample that sPMCA did not generate a positive result for was a 2011 sample found to be IHC-positive in the RPLN only. sPMCA also identified an additional 18 IHC-negative samples as PrP^CWD^-positive.

**Figure 1:**
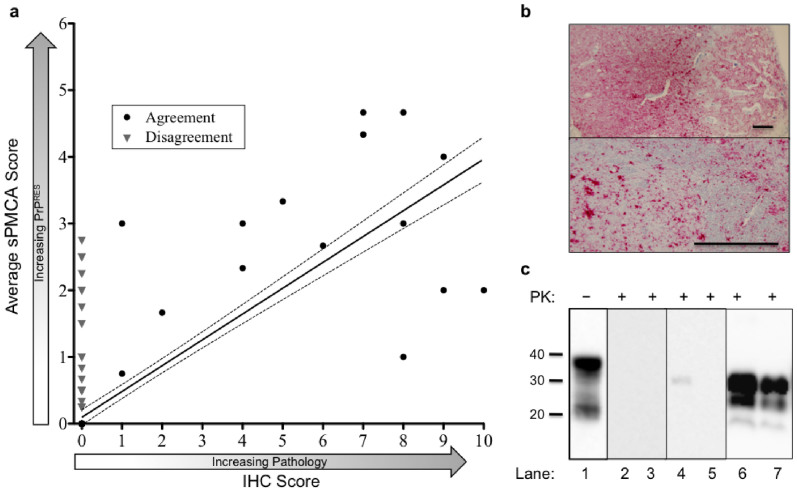
Correlation between IHC score and sPMCA score of each sample. (a) Sample scores by IHC and sPMCA were compared by linear regression to evaluate correlation between the two tests. Samples found positive by both tests are considered in “Agreement” (black circles). Samples found negative by IHC but positive by sPMCA are considered in “Disagreement (grey triangles). Each data point represents the mean of all replicates per animal. (b) Representative IHC of a positive obex sample. Scale bars, 100 μm. (c) Representative western blots: Lane 1, undigested NBH; lanes 2–3, negative elk obex from ND and MT after 6 rounds of sPMCA. Lanes 4–5, RMNP elk obex featured in (b) after 3 and after 6 sPMCA rounds (lanes 6 & 7).

We found a strong correlation between sPMCA and IHC scores for each elk sample (Figure 1). Linear regression found a positive association (slope = 0.39, R^2^ = 0.64) between samples scoring positive by both IHC and sPMCA. Samples that disagreed, scoring IHC negative but sPMCA positive, were not included in the linear regression, but are overlaid in Figure 1 to show the low sPMCA scores of samples that were otherwise IHC-negative. A high rate of sPMCA-positive samples in 2010 suggested an unidentified portion of those samples may have been false-positives due to contamination. This may also be the case for 2009. However, sPMCA did identify 4 additional positives in 2011 compared to the 3 found by IHC (6 total, Table 1 and Figure S1). One of the IHC-positive samples was the single disagreement mentioned above.

**Figure 2:**
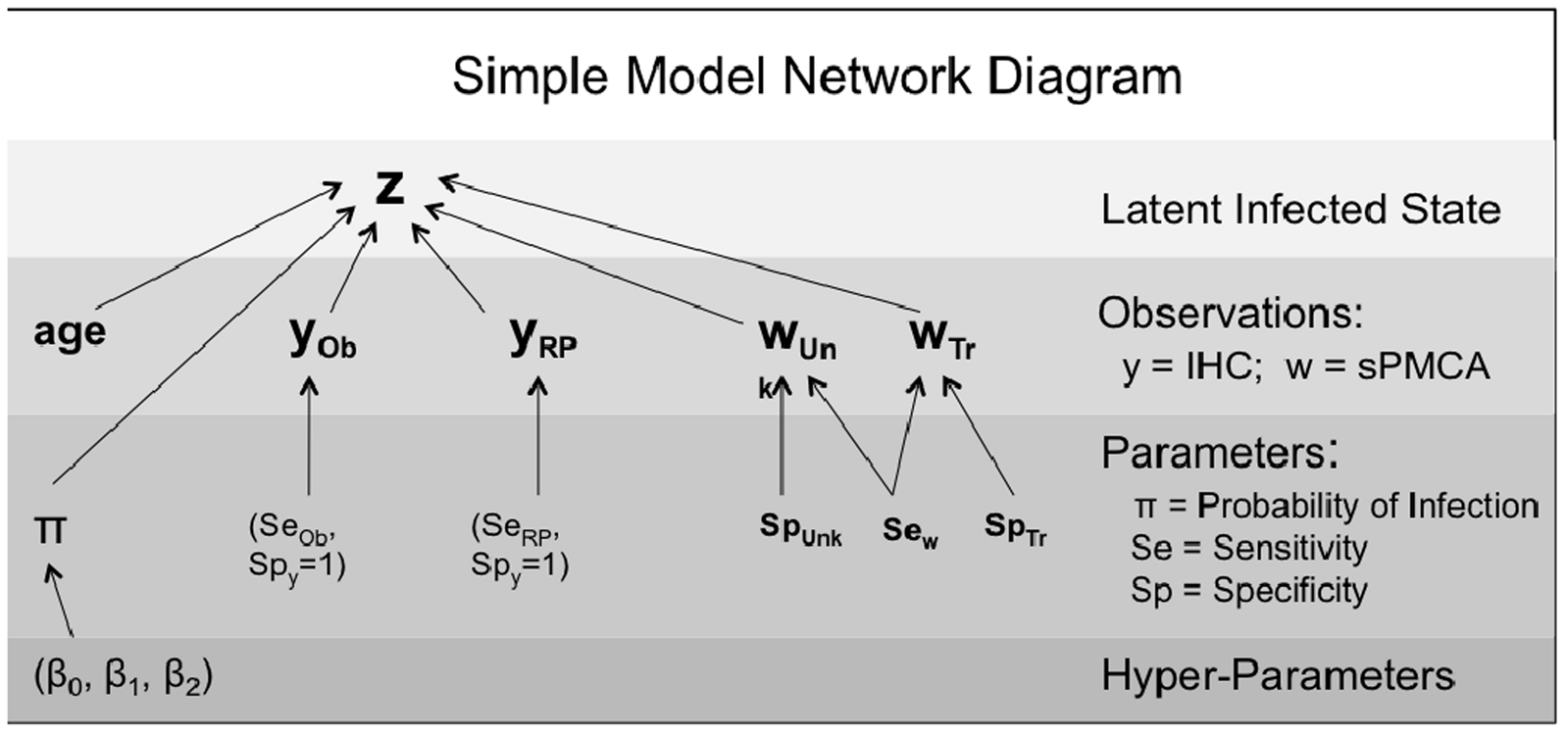
Model network diagram. Network diagram outlining the hierarchical Bayesian model used to estimate specificity and sensitivity of IHC and sPMCA and population prevalence of CWD. Ob, Obex; RP, Retropharyngeal lymph node; Unk, Unknown samples; Tr, Trusted samples.

We developed a novel hierarchical Bayesian model to estimate specificity, sensitivity and disease prevalence that considers all IHC and sPMCA test results (Figure 2). The model simultaneously estimates infection status of each individual animal and all derived quantities of interest, including estimates of PrP^CWD^ prevalence within the herd, and sensitivity and specificity of IHC for each tissue and sPMCA. This model also allowed us to quantify the potential effects of contamination in two of the collection years (2009 and 2010).

**Figure 3:**
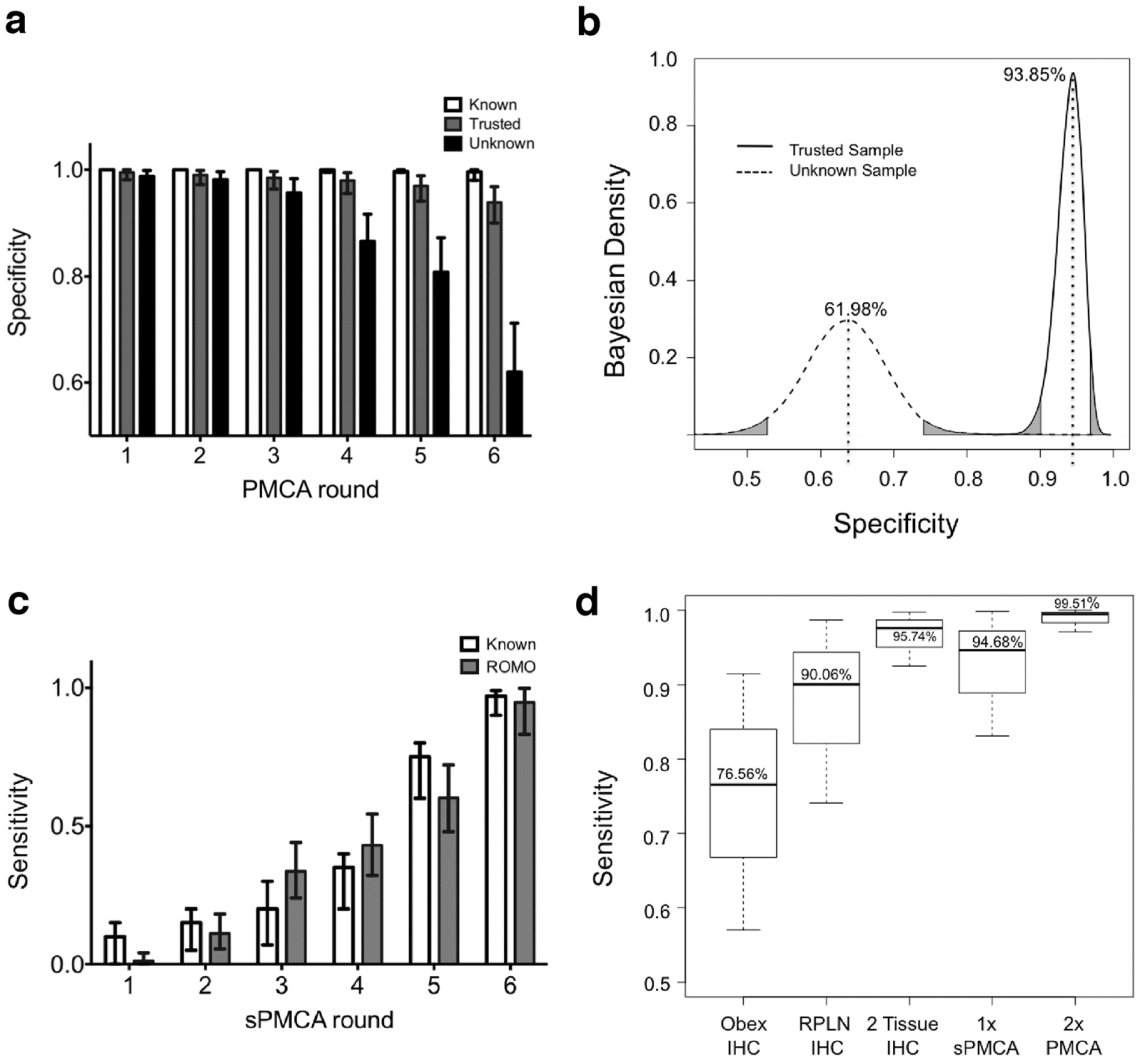
Specificity and sensitivity estimates of sPMCA and IHC. (a) Specificity estimates of sPMCA by amplification round. Three sample groups are presented: Known samples are positive controls used in previous studies and presented as raw data; Trusted samples are those which are verified by IHC or are from the 2011 sampling; and Unknown samples are all remaining samples which may include contamination-related false positives. (b) Overall sPMCA specificity estimates for Trusted and Unknown samples. The overall specificity of sPMCA is estimated at 93.9% (CI 90.1–96.9%) for Trusted samples and 63.4% (CI 52.6–74.1%) for Unknown samples. (c) Sensitivity of sPMCA by round. Sensitivity increases with each amplification round to 94.68% (CI 83.12–99.84%) by round 6. (d) Overall sensitivity estimates for IHC and sPMCA. Sensitivity estimates for IHC by individual tissue (obex (76.56%, CI 57.00–91.46%) and RPLN (90.06%, CI 74.13–98.70%)) and combined 98.99% (CI 90.01–100%); one replicate of sPMCA after 6 rounds (94.68%, CI 83.12–99.84%); and two replicates of sPMCA (99.51%, CI 97.15–100%).

The separate analysis of Trusted and Unknown samples allowed us to estimate sPMCA specificity by amplification round (Figure 3a) and for each group of samples (Figure 3b). When compared to specificity of raw data from previous sPMCA studies^23^,^35^,^39^ (99.60%, CI 98.00–100%) our model estimates specificity for our Trusted samples to be 93.85% (CI 90.00–96.84%), and a reduced specificity of 61.98% (CI 51.81–71.18%) for Unknown samples after six PMCA rounds.

We did not separate samples into Trusted and Unknown groups for sensitivity analyses because sensitivity is the ability of the test to correctly identify a true positive. The sensitivity of sPMCA after 6 rounds was estimated at 94.68% (CI 83.12–99.84%) for one replicate. Again we compared sensitivity of known positive samples (97.00%, confidence interval 95.07–99.02%) from previous work to demonstrate the agreement between raw data and the model estimates (Figure 3C). We typically assess at least two replicate samples by PMCA, which our model predicts to increase sensitivity to 99.51% (CI 97.15–100%) with corresponding decreases in specificity of trusted (91.03%, CI 84.42–97.80%) and unknown (42.01%, CI 27.72–50.11%) samples.

**Figure 4:**
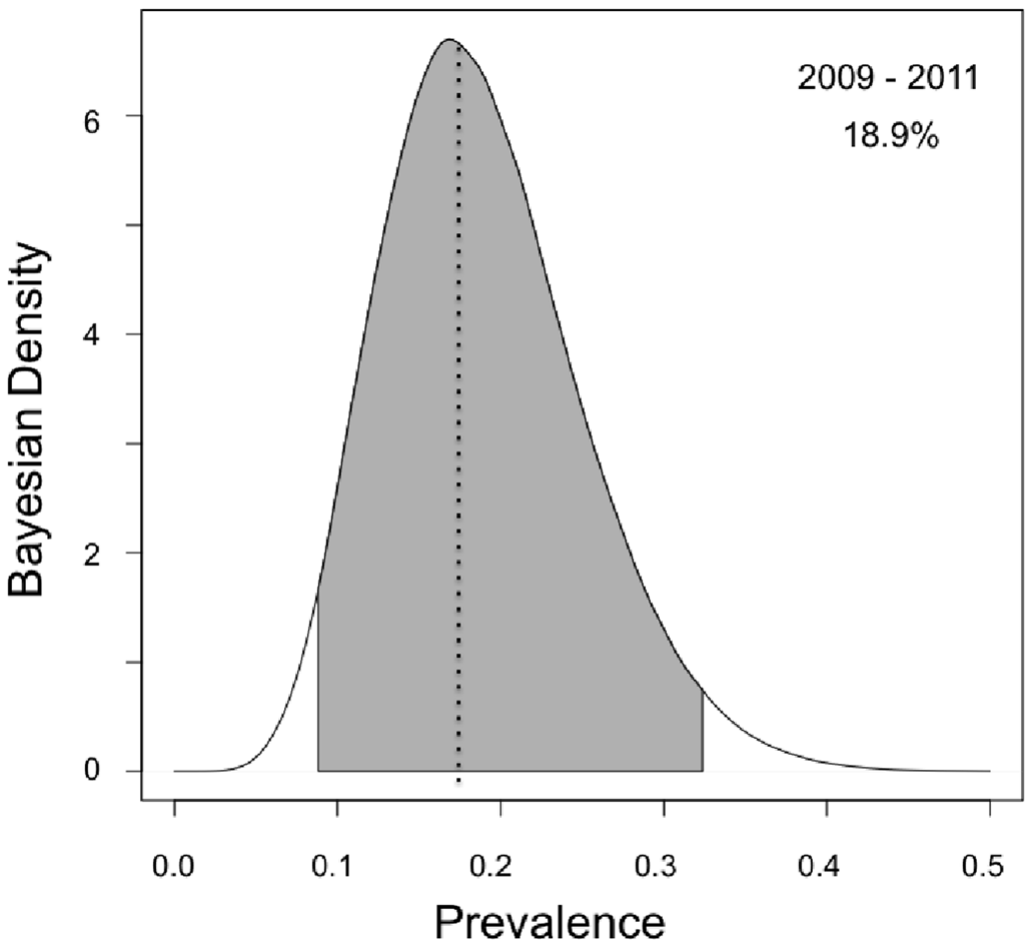
Overall prevalence estimate of prion infection for elk sampled in 2009-2011. The gray shaded area under the curve represents the 95% confidence interval.

We also calculated sensitivity estimates for IHC of obex and RPLN (Figure 3D). RPLN IHC (90.06%, CI 74.13–98.70%) was more sensitive than obex (76.56%, CI 57.00–91.46%). Combining obex and RPLN IHC increases sensitivity to 98.99% (CI 90.01–100%). Inputting all data from the two IHC and duplicate PMCA tests, our model estimated the overall period prevalence of CWD for 2009–2011 to be 18.90% (CI 8.71–32.39%; Figure 4).

**Figure 5:**
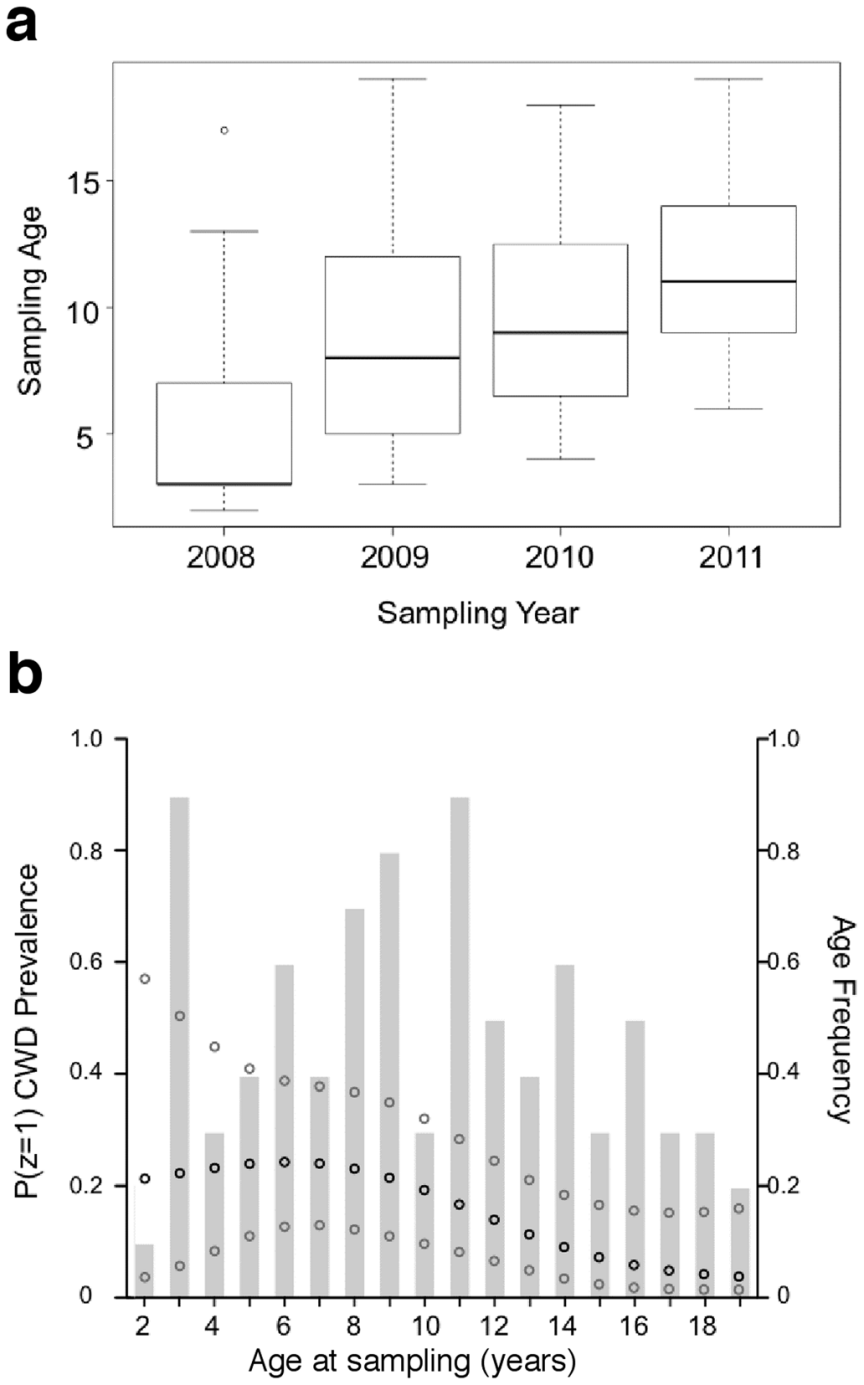
Age effect on prion infection prevalence. (a) Age distribution of animals sampled by year. (b) Derived posterior probability density for prevalence (P(z = 1)) across observed ages. Black dots represent mean values and gray dots, 95% confidence intervals. Left y-axis shows prevalence within age groups demonstrating the predominant age of infected animals lies between 2 and 10 years as previously documented. The right y-axis and the histogram in the background show the observed frequency of ages of elk sampled in this study.

The age distribution of elk sampled by year showed an overlap among years, with 2009 and 2010 including primarily middle-aged animals (Figure 5A). Our model predicts the primary age of infected animals lies between 2–11 years (Figure 5B). Means and credible intervals for the logistic regression coefficients, ®, are reported in Supplemental Table 2. No difference in overall prevalence was found between MM and ML elk (p = 1.0).

## Discussion

We compared specificity and sensitivity estimates of two tests for CWD prions in a free-ranging elk herd in RMNP, the gold standard PrP^CWD^ IHC versus sPMCA testing analyzed by Bayesian hierarchical modeling. We found that sPMCA detected PrP^CWD^ in the obex of infected elk with greater sensitivity than IHC of obex or RPLN. We used these estimates to predict prevalence of prion infection in this herd, and discovered animals with subclinical CWD may represent a significant carrier state in this population. Although there appears to be no upper age limit to infection in adult female elk^19^, we found that prevalence decreases with age and the primary age cohort for infection is 2 to 11 years.

sPMCA specificity was lower than expected, likely due to cross-contamination during necropsy in early years, which was reflected in the difference in sPMCA-positive results between 2010 and 2011. Our decontamination protocol instituted during necropsy in 2011 to limit cross-contamination reduced the sPMCA-positive rate to twice the IHC rate (sPMCA = 18.2% and IHC = 9.1%, Table 1). Our cross-contamination mitigation strategy provided us with a year of samples that we considered contamination-free.

We therefore consider the data showing the identification of 4 additional positive samples by sPMCA in 2011 to be reliable, and used them to inform our model of sPMCA specificity and sensitivity compared to IHC. Detection of four unique positives by sPMCA correlates with the low amounts of PrP^CWD^ predicted to exist in animals with early and sub-clinical disease.

Hierarchical Bayesian modeling allowed us to work with authentic but possibly imperfect data and let the model test parameters to find the best estimates. Having to discount some of the sPMCA findings for 2009 and 2010 was not ideal, but the model allowed us to adapt to the realities of research. When we removed possible false-positive results for specificity estimates the model estimated that specificity increased from 61.98% (Unknown samples) to 93.9% (Trusted samples). Uncertainty introduced by possible sample contamination likely resulted in the model predicting a lower specificity in these field samples than our previous specificity of 99.6% observed in controlled laboratory experiments^35^. Increased specificity using Trusted while excluding Unknown samples shows that sPMCA has a high specificity when cross-contamination is prevented. Moreover, if replicate samples are used, sensitivity increases to 99. 51%, while specificity remains acceptable at 91.03%. We consider these to be conservative estimates of sensitivity and specificity considering possible contamination at early collections, and our model allows us to use imperfect data to inform our model and estimate prevalence in this herd.

Our results suggest that prevalence of prion infection in this free-ranging RMNP elk herd is much higher than previously reported. Prior to 2013, the CWD prevalence in elk surrounding RMNP was estimated at <2%^19^,^40^,^41^. In 2013, Monello et al. reported CWD prevalence of 12.9% in RMNP based on PrP^CWD^ IHC of RAMALT^19^,^35^, over 4 times higher than previous reports. Here we report an estimated overall prevalence of 18.90%, consistent with the finding by Monello et al., who found 28% of adult female elk were infected during the course of their three-year study. We conclude that sPMCA can detect early cases of PrP^CWD^ infection and our model conservatively estimated overall prevalence since all known PrP^CWD^ positive animals from 2008, which tested positive on RAMALT biopsy, were removed from the sample population.

The higher overall prevalence estimate in this herd suggests previous measurements have been missing a large portion of PrP^CWD^ -positive animals and that a long history of exposure to prions and decades of relatively high densities on the winter range may have led to increased prevalence^19^,^42^,^43^. Further study is required to identify possible ecological differences in this herd compared to neighboring ones.

As an amplifying assay, sPMCA has previously been shown to be extremely specific and sensitive in prion detection studies^19^,^22^,^40^,^44^,^45^ but had not been directly compared to IHC in elk or in samples from free-ranging animals. This study has shown that sPMCA on the obex alone is more sensitive than IHC on obex or RPLN. sPMCA also detected several positive obex samples, which were IHC-negative from 2011. We argue that this increased detection represents early stage infections or sub-clinical animals, which may or may not shed PrP^CWD^ or develop clinical disease at a later time point.

Similar to Monello et al.^19^, our sensitivity analysis of IHC by tissue indicates that in this study population, that IHC in the RPLN was actually more effective in detecting positives animals than the obex. These results indicate that IHC on the obex might not be the best method to detect nascent PrP^CWD^ in elk, and perhaps the premise that the infection course is different between deer and elk is not absolute. Determining whether sPMCA in the RPLN would show a similar improvement on sensitivity compared to obex requires further study.

Our data demonstrate that previous IHC-based studies are possibly missing early stage or sub-clinical cases in sampled populations. It is widely accepted that IHC is sensitive enough to detect pre-clinical cases, but we propose that sPMCA can detect additional cases even earlier, possibly soon after infection. In previous work we found sPMCA had a detection limit of 10^−9^ 35,46 which is much more sensitive than the sensitivity of a mouse bioassay at 10^−4^. This suggests that animals found positive by sPMCA have much lower levels of PrP^CWD^ than animals with clinical disease, but are indeed infected. The detection of very early sub-clinical cases raises the question of biological relevance at the population level. We propose that this sub-clinical subset of the population may be ecologically important to the disease transmission cycle because of potential preclinical vertical transmission from mother to offspring^47^, horizontal transmission through direct contact, or indirect transmission through environmental deposits of prions.

It remains unclear when animals begin shedding prions into the environment. Through the use of a mouse bioassay Tamguney et al.^22^,^24^,^44^,^45^ showed asymptomatic deer were capable of shedding infectious levels of CWD as early as 10 months prior to clinical disease. Bioassays, both in mice and deer, have limited sensitivity so shedding could be occurring much earlier than 10 months post-infection but at levels insufficient to cause clinical disease in the infected host. It is also unclear if genotype plays a role in prion shedding, as well as disease course. Our data suggest that having at least one L allele at codon 132 does not alter the disease prevalence within the ML genotype, supporting data reported by Perucchini et al.^27^. The slow disease course and the potential existence of a carrier state facilitate a high prevalence and frequent opportunity for transmission between animals with the MM and ML genotypes.

It is commonly stated in the literature that CWD is an invariably fatal disease, but it may be more accurate to state that once animals begin to show clinical signs they are certain to succumb to CWD or other associated causes of death such as predation^4^,^24^,^48^. Perhaps other carrier states exist within the population, which may or may not contribute to the transmission and deposition of prions in the population and the environment. Further research is required to address the role of a carrier state in the ecology of CWD transmission.

The application of sPMCA will be important both to research and for diagnostic investigation, and may improve state and federal surveillance programs for CWD in both naïve and endemic host populations. Increased sensitivity, and the need for only obex tissue, may lead to detection of new focal points prior to clinical disease emerging in otherwise CWD-free populations. Additionally, in the economically and politically difficult scenario of culling captive herds that tested positive for CWD, extremely sensitive assays such as sPMCA of prions from tissue and excreta are essential to verify that more animals besides the index case were infected, and if any sub-clinical carriers may have been shedding into the environment.

Overall, our data contribute to the increasing evidence that a portion of a herd may be infected, but die from other causes while infected with PrP^CWD^ because of age, genetic susceptibility or other unknown factors. However, the contribution of prions shed into the environment from this sub-clinical population may be important and requires further investigation. The existence of an infectious PrP^CWD^ carrier state aligns with disease ecology theory, which proposes balance between transmissibility and pathogenesis of a pathogen. As such, through selection pressures from the host and external environment the pathogen will tend towards the greatest transmissibility strategy. CWD transmission may be more complicated than disease ecology might predict, since prolonged persistence and indirect transmission of prions in the environment may potentiate spread without affecting pathogenesis.

Despite the fact that prions are only protein, studies continue to point at evolutionary behavior and selection pressures of prions which indicate that like other pathogens, prions are capable of evolving and adapting to their environment^4^,^27^,^48^,^49^. With increasing prevalence at the population level, as is reported in this study, sPMCA will continue to be an important tool to investigate CWD in wildlife.

## Methods

### Mice

All mice were bred and maintained at Lab Animal Resources, accredited by the Association for Assessment and Accreditation of Lab Animal Care International, in accordance with protocols approved by the Institutional Animal Care and Use Committee at Colorado State University.

### Elk brain tissue samples

Brain tissues were collected at necropsy from 85 free-ranging elk that were radio collared and later euthanized for research and management purposes^19^. Briefly, 136 elk were initially captured, sampled and collared in 2008. Rectoanal mucosal-associated lymphatic tissue (RAMALT) samples were collected from each elk during initial capture and tested for PrP^CWD^ by IHC^7^,^8^,^9^,^38^,^50^. In 2008, samples were collected from 11 PrP^CWD^ -positive animals that were recaptured, euthanized and necropsied within two months of original capture. In subsequent years 17, 24, and 33 randomly selected animals were recaptured, euthanized, necropsied, and included in this study. These opportunistic collections were IHC-negative via RAMALT biopsy in 2008 and no elk exhibited clinical evidence of CWD when euthanized. Elk were euthanized in the field then transported to the TSE necropsy facility at the Colorado State University Veterinary Teaching Hospital within 8 hours of euthanasia^19^. We removed a primary incisor from all elk carcasses to determine age by cementum analysis (Matson Lab, Milltown, MT). Field euthanasia and subsequent necropsies were approved by NPS (permit ROMO-2007-SCI-0077), Colorado Division of Wildlife (permit TR1081), and CSU IACUC (permit 07-231A).

Multiple tissues were collected from each animal during necropsy. Here we compare IHC results from obex and retropharyngeal lymph nodes^14^,^15^,^50^,^51^ to sPMCA results from the obex alone. All lymph node samples and half the obex sample were fixed in 10% neutral buffered formalin for IHC analaysis; the other half of the obex sample was stored in a whirl pack at −80°C for testing by sPMCA.

### IHC

Sections of retropharyngeal lymph node (RPLN) and obex were examined by IHC as previously described^16^,^50^. Briefly for IHC, tissues were fixed, paraffin-embedded and 10 μm sections cut, mounted on glass microscope slides, and immunolabeled with anti-prion protein monoclonal antibodies (mAbs) F99/97.6.1 (mAb 99) and mAb P4. PrP^CWD^ was visualized by incubation with alkaline phosphatase (AP)-conjugated donkey antibodies against mouse IgG and fast red chromogen that revealed red aggregate deposits in neural and lymphoid tissues. A scoring system (0–10) was used to evaluate intensity of PrP^CWD^ deposition as described in Spraker et al.^52^.

### Brain Homogenization

Frozen elk obex samples were partially thawed and approximately 200 mg of tissue was collected from the interior of the obex sample, placed into a 2 ml tube containing silica beads and 180 μl of sPMCA buffer #1 (150 mM NaCl, 4 mM EDTA, in PBS) was added. Tissues were homogenized using a FastPrep machine (Thermo Scientific) as outlined in Meyerett et al.^46^. The clarified 10% homogenate supernatant was removed and stored at −80°C.

sPMCA substrate consisted of 10% mouse normal brain homogenate (NBH) prepared in a prion-free room from Tg5037 mice expressing cervid PrP^C^ as previously described^46^.

### sPMCA and western blotting

Twenty-five μl of RMNP elk obex homogenate was added to 25 μl NBH in 0.2 ml tubes. Samples were sonicated in a Misonix 4000 sonicator (Misonix Inc., Farmingdale, NY) for 40 s every 30 minutes for 24 hours at 37°C constituting one round^46^. For each subsequent round, 25 μl of each sample from the previous round was combined with 25 μl of fresh NBH. Duplicate samples were run for 6 sPMCA rounds to balance desired sensitivity and specificity (>90%) as previously observed^35^. Each sPMCA experiment contained at least six NBH-negative controls and two positive plate controls (CWD-positive elk brain homogenate E2, 1:1000). The negative brain samples were collected from Montana (n = 1) and North Dakota (n = 10) and homogenized as previously described^23^ Montana eNBH was confirmed negative by bioassay in CWD susceptible mice (data not shown) and ND samples were confirmed negative by ELISA (Bio-Rad Laboratories). Elk samples collected in 2008 and 2009 were not blind to laboratory researchers, but all 2010 and 2011 samples were blinded. The PrP^CWD^ status of all negative controls was known at the time of sPMCA.

PMCA samples were assayed by western blot as previously described^23^,^46^. Briefly, 18 μl of sample was digested with 2 μl of 50 μg/ml proteinase K (PK, Roche, Basel, Switzerland) for 30 minutes at 45°C. Samples were electrophoresed, electro-transferred to PVDF membranes and visualized with HRP-conjugated anti-PrP Bar-224 monoclonal antibody (SPI-Bio, Montigny-le-Bretonneux, France). Because prion load inversely correlates with the round in which it is first detected, samples were given a score inversely proportional to the first positive sPMCA round^23^. For example, if a sample scored positive in the first of six rounds, it received a sample score of 6. A positive sample detected in round six would receive a score of 1 and a negative sample would be scored 0.

### Cross-contamination management

During the first three years of necropsies (2008–2010), decontamination was not standard practice during tissue collection. Necropsies from 2009–2011 were performed in a new TSE necropsy room, reducing the risk of contamination. In 2011, a decontamination protocol was implemented to further prevent cross-contamination during sample collection, including sodium dodecyl sulfate/acetic acid^46^,^53^ treatment of working surfaces and all necropsy instruments, and glove and apron changes between animals. Disposable sterile scalpels were used for all CNS tissue harvest. Samples were processed according to protocols implemented to prevent cross-contamination at the lab bench, including using sterile scalpels, forceps and clean gloves during sub-sampling, homogenization and sPMCA.

### Model to estimate specificity, sensitivity and prevalence

The model is represented as a network diagram in Figure 2. Each animal was considered to have a true infection status, denoted *z_i_*, where *z_i_* = 0 when animal *i* is uninfected and *z_i_* = 1 when infected. Infection status was modeled as a logistic regression with age and age covariates^2^, as well as a categorical covariate for the samples from the year 2008, such that:
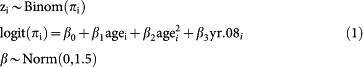
For example, the IHC results for the obex tissue were described by a mixture model as follows:
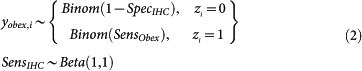
We denote sPMCA results for individual *i* and replicate *j* across amplification rounds *t* = *1:6* as *w_i,j,t_* which is contingent upon the latent disease state of individual *i*, Sensitivity and Specificity probabilities across amplification rounds, ***Se*** and ***Sp***, and the result from the previous round where applicable, *w_i,j,t-1_*.
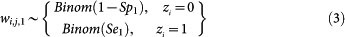

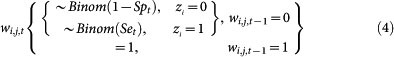


*Se* and *Sp* represent the probability of a sPMCA test result transitioning from a negative test result to a positive within amplification round *t*. These parameters are modeled as flat Dirichlet distributions of length *T + 1*. Incorporating the probability of a negative test result allows the probabilities to sum to one. This transition model is a modification of the Cormack-Jolly-Seber survival model with perfect detection^54^. The parallel occurs because after a sample is positive in one sPMCA cycle, it remains positive in the following rounds, just as, for example, a mortality event at any time guarantees all following times maintain that state. The model was fit to the data in JAGS 3.1.0^55^,^56^ with the rjags package^55^ in the R 2.15.1 computing environment^57^.

Errors in specificity, or false positives, can occur as a result of cross-contamination of samples during necropsy or possibly by spontaneous misfolding during sPMCA. We previously reported our method of sPMCA has a specificity of 99.6% in the laboratory setting^35^. Negative samples used for this sPMCA experiment were used to show specificity in our laboratory setting, but do not account for possible necropsy contamination of the elk tissues. To remove bias from possible necropsy-related false positives in years 2009 and 2010 we separated “Trusted” from “Unknown” samples. Trusted samples were those found positive by IHC (2008–2010) and all samples from 2011, when we employed decontamination techniques at necropsy. Unknown samples are IHC-negative samples from 2009 and 2010. sPMCA results for these samples could be true, sub-clinical positives outside of the detection limit of IHC, or they could be false positives resulting from contamination during sample collection at necropsy. To maintain a conservative estimate of the specificity of sPMCA, Trusted and Unknown samples were assumed to have independent specificity probabilities.

Errors in sensitivity, or false negatives, for either assay occurred for two reasons: either the concentration of PrP^CWD^ was below the detection limit of the assay or, despite the overall presence of PrP^CWD^ in the tissue, the exacted portion that was assayed did not contain detectable levels of PrP^CWD^ due to non-homogenous distribution^19^,^30^,^41^,^58^. All estimates are reported with a 95% Bayesian credible interval (CI).

Genetic data for 30 MM and 30 ML randomly selected animals were used to assess for prevalence differences between genotypes. IHC and sPMCA results were pooled allowing for positive or negative status to be tested against MM and ML genotype status using a Fisher's Exact Test (p < 0.05).

## Supplementary Material

Supplementary Information Supplementary Information

## Figures and Tables

**Table 1:** Summary of detection assay results

Sampling Year	*n* = elk sampled	sPMCA +	IHC +			
			*Obex*	*RPLN*	*Ob & RP*^a^	IHC + Total
2008^b^	11	11			11	11
2009	17	5^c^			1	1
2010	24	17^c^	1	3	1	5
2011^d^	33	6^e^		1	2	3
Totals	85	39	1	4	15	20

^a^PrP^CWD^ was found in both obex and RPLN samples.

^b^all animals were RAMALT-positive at start of study, euthanized in the field within two months and tested further.

^c^Cross-contamination of tissue samples at necropsy likely resulted in some false sPMCA-positives these years.

^d^A decontamination protocol was put in place to prevent cross-contamination at necropsy.

^e^sPMCA identified 4 additional samples that were positive for PrP^CWD^, but misdiagnosed one that was positive via IHC in RPLN only (the only such misdiagnosis by sPMCA).
