# Supplementary material for: Prion Amplification and Hierarchical Bayesian Modeling Refine Detection of Prion Infection
Source: Sci Rep. 2015 Feb 10;5:8358. doi: 10.1038/srep08358 (PMC5389033; doi:10.1038/srep08358)
Supplement: Supplementary Information [file srep08358-s1.pdf]

Prion Amplification and Hierarchical Bayesian Modeling Refine Detection of Prion Infection

A. Christy Wyckoff[1,2], Nathan Galloway[3], Crystal Meyerette-Reid[1], Jenny Powers[4], Terry Spraker[1], Ryan J. Monello[4], Bruce Pulford[1], Margaret Wild[4], Michael Antolin[3], Kurt VerCauteren[2] and Mark Zabel[1,*]

Supplementary Information

Hierarchical Bayesian Analysis

Hierarchical modeling is a powerful statistical tool that allows researchers to formulate flexible probability models to best represent the system giving rise to available data. We used several parameter levels to build our hierarchical model: true infection status, age, sensitivity and specificity for each teat for each tissue, and probability of infection (figure 1).

Bayesian application of these models takes advantage of previous knowledge of the state of the system being modeled, usually in the form of data derived from prior measurements or observations. For example, the probability of drawing an ace from a deck of cards is 1/52. But if five cards are drawn and none of them are aces, the probability of drawing an ace is now 1/47. Thus, previous data eliminates five cards from the deck, so that we can refine our prediction of drawing an ace on the next card. In our model, the data informing our model to improve our prediction are IHC and sPMCA test results and animal ages.

We can then use computational tools to fit these complex models to data and make scientific inference. This approach offers several benefits to researchers including but not limited to appropriately addressing uncertainty while incorporating multiple data sources in a flexible modeling framework. Uncertainty can be apportioned between observation error resulting from imperfect observation and process error inherent to the system. In our case, this imperfect data resulted from potential sample contamination in early collection years. All unknown parameters are treated as random and examined probabilistically. Inference is made not by null hypothesis significance testing as in deterministic statistics, but instead by examining the posterior distribution (and associated credible intervals) of the unknown parameters which result from consideration of all available data and the prior distributions representing pre-existing knowledge given the structure of the model. This approach is consistent with mathematical logic and can be used to make probabilistic predictions about the state of the world in a manner well suited to scientific inference and proved to be quite applicable to our research. Likelihood estimates in Bayesian models are expressed in probability distributions (or densities) rather than discrete probabilities. For further discussion, see McCarthy (2007), Royle and Dorazio (2008), and Hobbs and Hooten (In Press).

McCarthy M.A. 2007. Bayesian Methods for Ecology. *Cambridge University Press*, Cambridge.

Royle, J.A. And Dorazio, R.M. 2008. Hierarchical Modeling And Inference In Ecology: The Analysis Of Data From Populations, Metapopulations And Communities. Academic Press, San Diego, Ca.

Hobbs, N.T. and M.B. Hooten. (In Press). Bayesian Models: A Statistical Primer for Ecologists. *Princeton University Press*.

Supplementary Table 1: Summary statistics of posterior distributions of predicted prevalence of PrP^{CWD}-positive animals, π.

	Mean	0.025% CI	0.975% CI
Period	0.189	0.087	0.323
Annual 2009	0.311	0.217	0.409
Annual 2010	0.253	0.168	0.35
Annual 2011	0.18	0.104	0.277

Supplementary Table 2: Summary statistics of posterior distributions for β.

	Mean	0.025% CI	0.975% CI
β_0	-1.508	-2.349	-0.736
β_1	-0.791	-1.641	-0.040
β_2	-0.492	-1.368	0.281
b3,(2008)	3.812	2.105	5.708

Supplementary Table 3: Summary statistics of posterior distributions for sensitivity of IHC.

	Mean	0.025% CI	0.975% CI
Obex	0.766	0.570	0.915
RPLN	0.901	0.741	0.987
Full IHC	0.990	0.909	1.000

Supplementary Table 4: Summary statistics of posterior distributions for sPMCA test parameters.

	Mean	0.025% CI	0.975% CI
$1\text{-}\mathbf{Se}_7$	0.944	0.822	0.998
$\mathbf{Sp}_{Tr,7}$	0.939	0.901	0.969
$\mathbf{Sp}_{Unk,7}$	0.634	0.526	0.741

Supplementary Table 5: Summary statistics of posterior distributions for complete sPMCA sensitivity, **Se**$_{w}$, across amplification cycles.

	Mean	0.025% CI	0.975% CI
Neg	0.053	0.002	0.169
1	0.011	0.000	0.041
2	0.100	0.048	0.167
3	0.225	0.144	0.317
4	0.094	0.036	0.172
5	0.171	0.090	0.267
6	0.345	0.232	0.462

Supplementary Table 6: Summary statistics of posterior distributions for complete sPMCA specificity for trusted samples across amplification cycles, **Sp**$_{Tr}$.

	Mean	0.025% CI	0.975% CI
Neg	0.939	0.901	0.969
1	0.005	0.000	0.019
2	0.005	0.000	0.019
3	0.005	0.000	0.019
4	0.005	0.000	0.019
5	0.010	0.001	0.028
6	0.030	0.011	0.059

Supplementary Table 7: Summary statistics of posterior distributions for complete sPMCA specificity for unknown samples across amplification cycles, **Sp**$_{Unk}$.

	Mean	0.025% CI	0.975% CI
Neg	0.634	0.526	0.741
1	0.013	0.001	0.037
2	0.007	0.000	0.026
3	0.024	0.004	0.056
4	0.092	0.047	0.146
5	0.058	0.023	0.104
6	0.172	0.099	0.247

Supplementary Table 8: Summary statistics of posterior distributions for sPMCA sensitivity $(P(y>0 \mid z=1))$ with varying numbers of replicates.

# Replicates	Mean	0.025% CI	0.975% CI
Single	0.944	0.824	0.998
Double	0.995	0.969	1.000
Triple	0.999	0.995	1.000

Supplementary Table 9: Summary statistics of posterior distributions for sPMCA specificity $(P(y=0 \mid z=0))$ for trusted samples with varying numbers of replicates.

# Replicates	Mean	0.025% CI	0.975% CI
Single	0.939	0.900	0.969
Double	0.883	0.811	0.939
Triple	0.829	0.730	0.909

Supplementary Table 10: Summary statistics of posterior distributions for sPMCA specificity $(P(y=0 \mid z=0))$ for unknown samples with varying numbers of replicates.

# Replicates	Mean	0.025% CI	0.975% CI
Single	0.634	0.527	0.740
Double	0.405	0.278	0.548
Triple	0.260	0.147	0.405

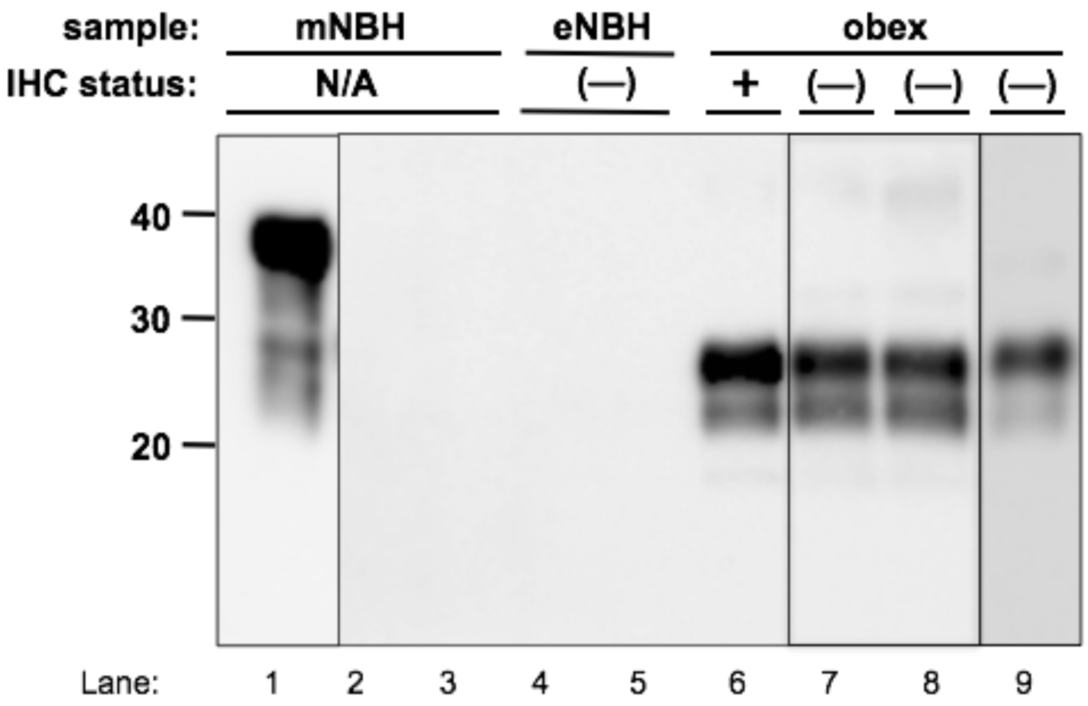

Figure S1. Representative western blot of select 2011 PMCA samples. All samples were digested with Proteinase K except the NBH sample in lane 1, which is shown as a shorter exposure of the adjacent blot for clarity. Murine NBH used as PMCA substrate (lanes 2-3) and elk NBH (lanes 4-5) serve as negative controls for PMCA. PMCA detected prions from one IHC+ obex (lane 6) and three IHC– samples (lanes 7-9). N/A, not applicable.
